# Erythrocyte Methotrexate–Polyglutamate Concentrations in Pediatric Inflammatory Bowel Disease

**DOI:** 10.1093/ibd/izaf035

**Published:** 2025-02-21

**Authors:** Eva Vermeer, Eduard A Struys, Marry Lin, Johan E van Limbergen, Nanne K H de Boer, Maja Bulatović-Ćalasan, Tim G J de Meij, Robert de Jonge

**Affiliations:** Department of Paediatric Gastroenterology, Emma Children’s Hospital, Amsterdam University Medical Centre, Amsterdam, The Netherlands; Amsterdam Gastroenterology Endocrinology Metabolism (AGEM) Research Institute, Amsterdam University Medical Centre, Amsterdam, The Netherlands; Amsterdam Reproduction & Development (AR&D) Research Institute, Amsterdam University Medical Centre, Amsterdam, The Netherlands; Laboratory of Specialised Diagnostics and Research, Department of Laboratory Medicine, Amsterdam University Medical Centre, Amsterdam, The Netherlands; Laboratory of Specialised Diagnostics and Research, Department of Laboratory Medicine, Amsterdam University Medical Centre, Amsterdam, The Netherlands; Department of Paediatric Gastroenterology, Emma Children’s Hospital, Amsterdam University Medical Centre, Amsterdam, The Netherlands; Amsterdam Gastroenterology Endocrinology Metabolism (AGEM) Research Institute, Amsterdam University Medical Centre, Amsterdam, The Netherlands; Amsterdam Reproduction & Development (AR&D) Research Institute, Amsterdam University Medical Centre, Amsterdam, The Netherlands; Amsterdam Gastroenterology Endocrinology Metabolism (AGEM) Research Institute, Amsterdam University Medical Centre, Amsterdam, The Netherlands; Department of Gastroenterology and Hepatology, Amsterdam University Medical Centre, Amsterdam, The Netherlands; Laboratory of Specialised Diagnostics and Research, Department of Laboratory Medicine, Amsterdam University Medical Centre, Amsterdam, The Netherlands; Department of Rheumatology and Clinical Immunology, University Medical Centre Utrecht, Utrecht, The Netherlands; Department of Paediatric Gastroenterology, Emma Children’s Hospital, Amsterdam University Medical Centre, Amsterdam, The Netherlands; Amsterdam Gastroenterology Endocrinology Metabolism (AGEM) Research Institute, Amsterdam University Medical Centre, Amsterdam, The Netherlands; Amsterdam Reproduction & Development (AR&D) Research Institute, Amsterdam University Medical Centre, Amsterdam, The Netherlands; Laboratory of Specialised Diagnostics and Research, Department of Laboratory Medicine, Amsterdam University Medical Centre, Amsterdam, The Netherlands

**Keywords:** pediatric inflammatory bowel disease, Crohn’s disease, ulcerative colitis, methotrexate, therapeutic drug monitoring

## Abstract

**Background and Aims:**

Therapeutic drug monitoring (TDM) of methotrexate (MTX) is challenging due to its pharmacokinetics and short plasma half-life. Intracellular MTX–polyglutamates (PG_1–5_), which accumulate over time, have not been assessed in pediatric inflammatory bowel disease (IBD). This study aimed to evaluate erythrocyte MTX-PG as a potential TDM tool in pediatric IBD.

**Methods:**

In this cross-sectional study, MTX-PG concentrations were measured in erythrocytes of children with IBD on stable low-dose MTX for at least 12 weeks using stable-isotope dilution liquid chromatography–tandem mass spectrometry. The influence of administration route, MTX dosage, and anthropometrics on MTX-PG concentrations was examined.

**Results:**

Seventy-eight patients were included, showing MTX-PG_3_ as the predominant subspecies (median 27.0 nmol/L) with a median MTX-PG_total_ of 74.8 nmol/L. A higher MTX dose correlated significantly with elevated levels of MTX-PG_3_, MTX-PG_4_, MTX-PG_5_, and MTX-PG_total_ (*P* < .01). Adjusted for body surface area, MTX dose remained significantly associated with higher MTX-PG concentrations (*P* < .01). However, comparison by administration route was limited due to a few patients on subcutaneous MTX (*n* = 4).

**Conclusions:**

We observed high interindividual variability in the reached erythrocyte MTX-PG concentrations. Body surface adjusted or unadjusted MTX dosage showed a positive linear correlation with erythrocyte MTX-PG concentrations in children with IBD. This is a prerequisite for TDM and provides a strong basis for further research into the relation between TDM of MTX, efficacy, and toxicity.

**Key messages**

**What is already known?**

- Methotrexate–polyglutamates (MTX-PGs) have shown promise as a therapeutic drug monitoring (TDM) tool in various immune-mediated inflammatory diseases, but data in pediatric inflammatory bowel disease (IBD) are lacking.

**What is new here?**

- This study established that MTX-PGs are measurable in pediatric IBD, and dose-dependent variations and significant interindividual variability were observed, highlighting the potential of MTX-PG monitoring as a TDM tool in pediatric IBD.

**How can this study help patient care?**

- Implementing MTX-PG monitoring can improve individualized dosing strategies, enhancing therapeutic efficacy and minimizing adverse effects in pediatric IBD management.

## Introduction

Inflammatory bowel disease (IBD) is a chronic condition characterized by relapsing inflammation of the intestines and encompasses Crohn’s disease (CD), ulcerative colitis (UC), and IBD-unclassified (IBDU). In the general population, the prevalence of IBD is approaching 1% and it is estimated that a total of 15% of cases present before the age of 18 years.^1,2^ Recent epidemiological studies also show an increase in pediatric IBD both globally and in the Dutch population, associated with considerable morbidity and healthcare costs.^3,4^

Main goals in the treatment of pediatric IBD are to achieve clinical remission through the reduction of inflammation, optimize growth and development, improve quality of life, and promote mucosal healing while minimizing drug toxicity, in line with a treat-to-target approach.^5^ Potential maintenance treatment options are nutritional therapy, 5-aminosalicylic acid (5-ASA) compounds, methotrexate (MTX), thiopurines, tumor necrosis factor alpha inhibitors (anti-TNFα) such as infliximab (IFX), or other biologicals such as ustekinumab and vedolizumab.^5,6^ In recent years, MTX has gained popularity in pediatric IBD due to increasing concerns of malignancy, infection, and risk of hemophagocytic lymphohistiocystosis in thiopurine therapy.^7–10^ MTX is recommended as a first-choice immunomodulator for maintenance of remission, or after thiopurine failure or intolerance, as well as in combination with a biological to prevent antidrug antibody (ADA) development in pediatric CD by the recent ECCO/ESPGHAN guidelines.^5,11^

MTX is a folate antagonist with anti-inflammatory, immunomodulatory, and anti-proliferative effects. High-dose MTX (up to 500 mg/m^2^) is widely used in several neoplastic diseases, such as leukemia, non-Hodgkin lymphoma, and osteosarcoma.^12^ Low-dose MTX (up to 25 mg/week) is prescribed for various immune-mediated inflammatory diseases (IMIDs), such as rheumatoid arthritis, juvenile idiopathic arthritis, atopic dermatitis, psoriasis, sarcoidosis, and CD.^13–15^ Circulating MTX in its monoglutamate form (MTX-PG_1_) is rapidly transported into (hematopoietic) cells by the reduced folate carrier.^16^ Subsequently, polyglutamylation takes place intracellularly, during which the enzyme folylpolyglutamate synthetase (FPGS) adds glutamate moieties to the native MTX-PG_1_ and forms MTX-PG_2-6_. A higher degree of polyglutamylation enhances cellular retention of MTX, as longer MTX-PG chains are not easily expelled by the efflux transporters, which prolongs MTX’s presence in the cell.^17–19^ Concurrently, polyglutamylation increases MTX’s efficacy through stronger inhibition of target enzymes within the folate, pyrimidine, and purine pathways, leading to MTX’s anti-proliferative effects in leukemia. Additionally, MTX’s sustained activity promotes the accumulation of anti-inflammatory adenosine, believed to mediate its therapeutic effects in IMIDs.

In (pediatric) IBD, therapeutic drug monitoring (TDM) is available and implemented in clinical practice for thiopurines and biologics. TDM encompasses the measurement of drug concentrations and subsequent optimization of dosing and/or administration route in order to achieve the aforementioned treatment goals, hereby improving clinical efficacy and minimizing adverse events.^20^ However, in MTX therapy, an effective TDM tool is lacking. The reason for this lies in MTX’s peculiar intracellular pharmacokinetics and its short half-life in plasma of approximately 6 hours.^21^ In previous work, our study group showed that erythrocyte MTX-PG measurements in low-dose therapy fulfills all requirements for TDM, since there is a reliable and validated method available to measure intracellular concentrations, large interindividual variation in drug concentrations, stable intraindividual drug levels, and a concentration–effect relationship.^22–26^ Hence, we proposed that erythrocyte MTX-PGs deserved further study as a TDM tool in pediatric IBD.

In this observational cross-sectional study, we aimed to determine erythrocyte MTX-PG concentrations in children with IBD during maintenance therapy. A secondary aim was to identify potential determinants of MTX-PG levels, such as sex, age, diagnosis, MTX dose, route of MTX administration, and duration of MTX treatment.

## Materials and Methods

### Study Design

Between February 2022 and April 2024, patients from the outpatient clinics and short-stay units of the Department of Paediatric Gastroenterology at Amsterdam University Medical Centre were recruited for this cross-sectional study. All patients younger than 18 years, diagnosed with IBD according to the revised Porto criteria, and using low-dose MTX maintenance therapy either orally or subcutaneously on a stable dose for at least 12 weeks, were eligible for inclusion.^27^ Low-dose MTX was defined as an absolute dose not exceeding 25 mg/week.

Demographic and clinical data were collected from the electronic patient files, including age, sex, height, weight, body surface area (BSA), diagnosis, MTX dose, route of MTX administration, duration of MTX therapy, comedication, Paediatric Crohn’s Disease Activity Index (PCDAI), Paediatric Ulcerative Colitis Activity Index, Paris classification, C-reactive protein (CRP), leucocyte count, aspartate aminotransferase (ASAT), alanine aminotransferase (ALAT), gamma-glutamyl transferase (γGT), and creatinine levels.

For the MTX-PG concentration measurements, 1 mL of whole blood was required and therefore residual material from routinely drawn venous EDTA blood sufficed. Because of this, participants did not need to undergo any additional blood withdrawals than the standard treatment protocol dictated. Since patients were recruited during their routine hospital visits, the timing of blood withdrawal within the weekly MTX schedule varied. To prevent this variation from affecting the results significantly, patients were enrolled after at least 12 weeks of MTX administration, since MTX-PG levels in erythrocytes plateau after approximately 12 weeks.^22,28^

### MTX-PG Analysis

EDTA whole-blood residual material was aliquoted and stored at −80 ^o^C until analysis. Individual MTX-PG concentrations were measured as described by Den Boer et al.^29^ In short, MTX-PGs were extracted from the samples by incubation with perchloric acid on ice. Upon centrifugation, the supernatant was filtered and subsequently analyzed through liquid chromatography–tandem mass spectrometry (LC-MS/MS) using stable-isotope labeled internal standards of MTX-PG_1-5_. MTX-PG concentrations were reported in nmol/L packed erythrocytes, and were corrected for hematocrit. MTX-PG_total_ is the sum of the individually quantified MTX-PG_1-5_ concentrations.

### Statistical Analysis

Normally distributed data were reported as means with standard deviations (SDs), while non-normally distributed data were presented as medians with interquartile ranges (IQRs). Variability of MTX-PG levels was depicted as the percentage coefficient of variation, defined as SD divided by the mean multiplied by 100%.

We performed correlation analyses for the dependent variables, that is, individual MTX-PG and MTX-PG_total_ concentrations, with the following independent variables: age, sex, length, weight, BSA, MTX dose, MTX dose per BSA, duration of MTX treatment, and route of administration. The Pearson correlation was used if both continuous variables were normally distributed, or in the case of 1 normally distributed continuous variable and 1 dichotomous variable. In the case of non-normally distributed data, the Spearman correlation was conducted.

A *P*-value <.05 was considered statistically significant, and all statistical analyses were performed using the Statistical Package for Social Sciences (IBM; version 28).

### Sample Size Calculation

A formal sample size calculation could not be performed because of the lack of available data in the field of pediatric IBD. We performed an explorative observational study and aimed to include approximately 80 subjects, which was comparable to a recent study on the use of MTX-PG concentrations as a TDM tool in adult CD patients.^30^

### Ethical Approval

This study was approved by the Medical Ethics Review Committee of VU University Medical Centre on February 17, 2022, under file number 2021.0780. Verbal consent was obtained from the participating children and/or their parents or guardians.

## Results

### Patient Characteristics

During the study period, we included a total of 78 children with IBD (68 CD, 6 UC, 4 IBDU) in the maintenance phase of their MTX treatment. The median duration of MTX therapy was 25.1 weeks, most patients used MTX orally (94.9%) and in combination with another immunosuppressant (80.1%), at a mean dose of 13.0 mg/week. All patients used 5 mg of folic acid per week, taken at least 24 hours after MTX administration, with the exception of 2 patients who used 10 mg of folic acid per week. Table 1 provides a detailed overview of the patient characteristics.

**Table 1.** Demographic and disease characteristics of the study population.

		All patients
Sex, *n* (%)	Female	28 (35.9)
	Male	50 (64.1)
Route, *n* (%)	Oral	74 (94.9)
	Subcutaneous	4 (5.1)
Diagnosis, *n* (%)	CD	68 (87.2)
	UC	6 (7.7)
	IBDU	4 (5.1)
Age (years), median (IQR)	*n* = 78	15.6 (13.4-16.8)
BSA (m^2^), mean ± SD	*n* = 78	1.5 ± 0.3
MTX dose (mg/week), mean ± SD	*n* = 78	13.0 ± 4.0
MTX dose/BSA (mg/week/m^2^), mean ± SD	*n* = 78	8.4 ± 2.0
Duration of MTX treatment (weeks), median (IQR)	*n* = 78	25.1 (17.3-34.4)
PCDAI, median (IQR)	*n* = 25	5.0 (0.0-5.5)
PUCAI, median (IQR)	*n* = 12	0.0 (0.0-0.0)
FCP (µg/g), median (IQR)	*n* = 54	140.5 (30.5-503.8)
CRP (mg/L), median (IQR)	*n* = 78	0.9 (0.6-3.4)
Leucocytes (10^9^/L), mean ± SD	*n* = 77	6.8 ± 2.0
ASAT (U/L), median (IQR)	*n* = 31	25.0 (19.0-29.0)
ALAT (U/L), median (IQR)	*n* = 72	19.5 (13.3-26.0)
γGT (U/L), median (IQR)	*n* = 70	13.0 (10.0-17.0)
Creatinine (µmol/L), median (IQR)	*n* = 51	60.0 (46.0-70.0)
Folic acid use, *n* (%)	*n* = 78	78 (100)
Concomitant medication, *n* (%)	*n* = 78	63 (80.1)
	Infliximab	58 (74.4)
	Mesalazine	3 (3.8)
	Budesonide	3 (3.8)
	Vedolizumab	2 (2.6)
	Adalimumab	2 (2.6)
Paris classification of CD, *n* (%*)*	*n* = 68	
Localization	L1: ileal	10 (14.7)
	L2: colonic	10 (14.7)
	L3: ileocolonic	50 (73.5)
	L4a: upper disease proximal to Ligament of Treitz	41 (60.3)
	L4b: upper disease distal to Ligament of Treitz and proximal to distal 1/3 ileum	12 (17.6)
Behavior	B1: nonstricturing, nonpenetrating	50 (73.5)
	B2: stricturing	8 (11.8)
	B3: penetrating	4 (5.9)
	B2B3: stricturing and penetrating	1 (1.5)
	p: perianal disease modifier	4 (5.9)
Paris classification of UC, *n* (%*)*	*n* = 10	
Extent	E1: ulcerative proctitis	1 (10.0)
	E2: left-sided UC (distal splenic flexure)	3 (30)
	E3: extensive (hepatic flexure distally)	2 (20)
	E4: pancolitis	4 (40)
Severity	S0: never severe	4 (40)
	S1: ever severe	6 (60)

Abbreviations: ASAT, aspartate aminotransferase; ALAT, alanine aminotransferase; γCD, Crohn’s disease; BSA, body surface area; CRP, C-reactive protein; FCP, fecal calprotectin; GT, gamma-glutamyl transferase; IBDU, inflammatory bowel disease—unclassified; MTX, methotrexate; PCDAI, Paediatric Crohn’s Disease Activity Index; PUCAI, Paediatric Ulcerative Colitis Activity Index; UC, ulcerative colitis.

### Erythrocyte MTX-PG Concentrations

**Figure 1.**
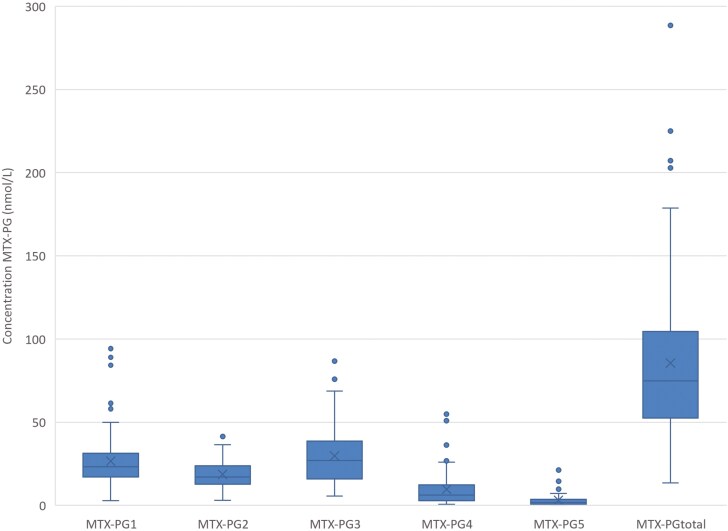
Erythrocyte MTX-PG concentrations in nmol/L packed erythrocyte. Box plot depicts median and IQ, cross depicts the mean. MTX-PG, methotrexate–polyglutamate.

All included patients had detectable MTX-PG_2_ and MTX-PG_3_ levels. MTX-PG_1_ was detected in 97.4% of subjects, and MTX-PG_4_ and MTX-PG_5_ were detected in 94.9% and 65.4% of patients, respectively. The predominant subspecies was MTX-PG_3_, with a median concentration of 27.0 nmol/L. The interindividual variation of the MTX-PG concentrations ranged between 41.3% and 113.8%. Details on MTX-PG concentrations are depicted in Table 2 and Figure 1.

**Table 2.** Median erythrocyte MTX-PG concentrations in nmol/L and their interindividual variations.

	*N* (%)	Median (IQR)	Coefficient of variation (%)
MTX-PG_1_	76 (97.4)	23.3 (17.1-31.3)	62.1
MTX-PG_2_	78 (100)	17.1 (12.8-23.8)	41.3
MTX-PG_3_	78 (100)	27.0 (15.9-38.7)	63.8
MTX-PG_4_	74 (94.9)	6.2 (2.9-12.3)	107.0
MTX-PG_5_	51 (65.4)	1.7 (0.7-3.6)	133.8
MTX-PG_total_	78 (100)	74.8 (52.6-104.6)	55.4

Abbreviations: IQR, interquartile range; MTX-PG, methotrexate–polyglutamate.

### Determinants of MTX-PG Concentrations

A higher dose of MTX correlated with significantly higher MTX-PG_3_ (*ρ* = 0.533, *P* < .01), MTX-PG_4_ (*ρ* = 0.522, *P* < .01), MTX-PG_5_ (*ρ* = 0.448, *P* < .01), and MTX-PG_total_ (*ρ* = 0.451, *P* < .01) concentrations. MTX dose explained 28.4%, 27.2%, 20.1%, and 20.4% of the variability (*R*^2^) of the MTX-PG_3_, MTX-PG_4_, MTX-PG_5_, and MTXPG_total_ concentrations, respectively.

**Figure 2.**
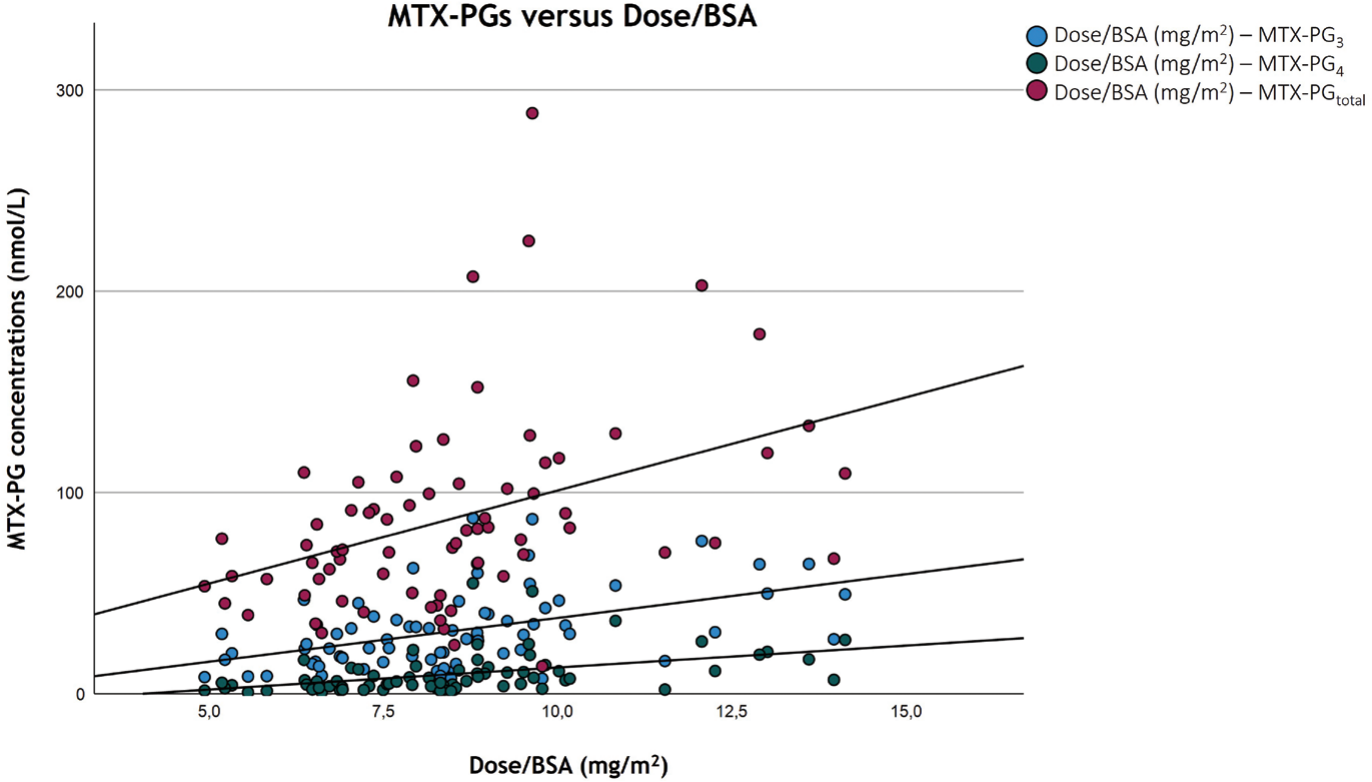
Correlations of erythrocyte MTX-PG concentrations with dosage adjusted for BSA. Only significant correlations are shown. BSA, body surface area; MTX-PG, methotrexate–polyglutamate.

When adjusted for BSA, higher MTX dose also correlated with higher MTX-PG_3_ (*ρ* = 0.475, *P* < .01), MTX-PG_4_ (*ρ* = 0.433, *P* < .01), and MTX-PG_total_ (*ρ* = 0.389, *P* < .01) concentrations. MTX dose per BSA explained 22.6%, 18.8%, and 15.1% of their variability of the MTX-PG_3_, MTX-PG_4_, and MTX-PG_total_ concentrations, respectively. Figure 2 depicts these correlations.

Weak positive correlations were found between age, weight, and BSA, and MTX-PG_3_, MTX-PG_4_, and MTX-PG_5_. We did not find statistically significant correlations between any of the individual or total MTX-PG concentrations and length, route of administration, or duration of treatment. Table S1 shows a detailed overview of correlation coefficients and their *P*-values.

## Discussion

In this study, we measured erythrocyte MTX-PG concentrations in a large cohort of children with IBD. Erythrocyte MTX-PG_3_ was shown to be the most abundant subspecies. A higher MTX dose correlated with higher longer-chain MTX-PG levels, also when the dose was adjusted for BSA. Moreover, we found high interindividual variability of the MTX-PG levels.

To our knowledge, only 1 other study has thus far reported measurements of erythrocyte MTX-PGs in pediatric IBD.^31^ That study included 21 children, all of whom were concurrently receiving infliximab, with 86% taking MTX orally and 14% taking MTX subcutaneously. MTX-PG concentrations were measured using high-performance liquid chromatography–tandem mass spectrometry (HPLC-MS/MS). Consistent with the presented findings, they observed a positive correlation between MTX dosage and both long-chain (MTX-PG_3-5_) and total MTX-PG concentrations, while no correlation was found with short-chain MTX-PGs (MTX-PG_1,2_). Furthermore, they reported significantly elevated levels of short-chain MTX-PGs in patients in clinical remission compared to those with active disease, despite no observed differences in infliximab levels. However, since all participants in the study by Morrow et al.^31^ were treated with a combination of MTX and infliximab, the therapeutic response could not be solely attributed to MTX. Importantly, Morrow et al.^31^ did not report on the duration of MTX treatment, a key factor in interpreting MTX-PG levels, nor did they differentiate between individual MTX-PG subspecies, focusing only on short- versus long-chain forms.

Van de Meeberg et al.^25^ recently conducted a cross-sectional study in adult CD. Using the same LC-MS/MS technique, these authors reported a high degree of interindividual variability in both individual MTX-PG and total MTX-PG concentrations, consistent with our findings. In contrast, they found that subcutaneous administration of MTX was associated with higher MTX-PG_4,5_ levels compared to oral administration, and increasing age was associated with elevated MTX-PG_total_ levels. Similar to our results, MTX-PG_3_ was the predominant subspecies, and the median MTX-PG_total_ was 117.1 nmol/L, closely resembling our median MTX-PG_total_ concentration of 101.9 nmol/L. The slightly higher values for MTX-PG_total_ concentrations in their adult cohort in comparison to the pediatric population in our study may be due to the fact that MTX-PG_total_ levels tend to increase with age. Additionally, variability in oral absorption and lower dosing in our cohort serve as important confounders.

Studies show that MTX-PG levels in adults with IBD vary significantly between individuals, with total MTX-PG concentrations ranging widely.^32–34^ While 1 study found no significant difference in MTX-PG levels between patients on 15 and 25 mg/week doses, others report that higher MTX doses result in increased levels of long-chain MTX-PGs, which is consistent with the literature in pediatric cohorts.^31,35^ This suggests a dose-dependent shift from short-chain to long-chain MTX-PGs, likely due to a substrate push effect rather than changes in FPGS enzyme activity.^23^ Despite not observing a reduction in short-chain MTX-PGs at higher doses in our current sample, we anticipate this effect would become more apparent with a larger sample size.

As mentioned, a limitation of this study is the relatively limited sample size. When divided into subgroups of disease, some determinants had a low prevalence in this study population, such as subcutaneous administration or other IBD phenotypes than CD. Given that only 2 subjects received subcutaneous MTX, the findings of our study are applicable primarily to those receiving oral administration. Furthermore, the study design does not allow for conclusions regarding the relationship between MTX-PGs and disease activity. Nonetheless, we observed that a higher dose of MTX is associated with higher long-chain MTX-PG concentrations, confirming previous reports in adult CD.^32–34^

To adequately evaluate the potential of MTX-PGs for TDM, more research is warranted. We would recommend exploring the correlation between MTX-PG concentrations and clinical response, as well as their association with adverse events attributed to MTX.

## Conclusions

In this study, MTX dosage showed a positive linear correlation with erythrocyte MTX-PG concentrations in children with IBD. The reached intracellular levels and their distribution profiles matched studies in adult IBD and other IMIDs. Furthermore, we observed high interindividual variability in the reached erythrocyte MTX-PG concentrations. Further research into concentration–effect and concentration–toxicity relationship is warranted in order to advance the use of TDM in MTX treatment of pediatric IBD.

## Supplementary Material

izaf035_Supplementary_Table_S1

## Data Availability

The data that support the findings of this study are available on request from the corresponding author.
