# Supplementary material for: Erythrocyte Methotrexate–Polyglutamate Concentrations in Pediatric Inflammatory Bowel Disease
Source: Inflamm Bowel Dis. 2025 Feb 21;31(9):2503–10. doi: 10.1093/ibd/izaf035 (PMC12455603; doi:10.1093/ibd/izaf035)
Supplement: izaf035_Supplementary_Table_S1 [file izaf035_supplementary_table_s1.docx]

**Supplementary material**

**Supplementary Table 1. Determinants of erythrocyte MTX-PG concentrations.**

Significant values are bold. * Correlation is significant with p < 0.05 (2-tailed). ** Correlation is significant with p < 0.01 (2-tailed).

Rho (ρ) represents the correlation coefficient, represented by the Pearson correlation coefficient for normally distributed continuous or dichotomous variables, or the Spearman's rank correlation coefficient for non-normally distributed continuous variables.

Beta (β) represents the standardised beta derived from univariable linear regression analysis.

R squared (R^2^) represents the explained variability derived from univariable linear regression analysis. It is only shown if the variable was significant at univariate analysis.

|  | MTX-PG_1_ | | | MTX-PG_2_ | | | MTX-PG_3_ | | | MTX-PG_4_ | | | MTX-PG_5_ | | | MTX-PG_total_ | | |
| --- | --- | --- | --- | --- | --- | --- | --- | --- | --- | --- | --- | --- | --- | --- | --- | --- | --- | --- |
|  | ρ | β | R^2^ | ρ | β | R^2^ | ρ | β | R^2^ | ρ | β | R^2^ | ρ | β | R^2^ | ρ | β | R^2^ |
| Age | -0,096 | -0,013 |  | 0,021 | 0,096 |  | **0,266*** | **0,280*** | **0,078*** | **0,286*** | **0,274*** | **0,075*** | **0,318*** | **0,305*** | **0,093*** | 0,182 | 0,230 |  |
| Sex | -0,062 | -0,062 |  | -0,177 | -0,177 |  | -0,084 | -0,084 |  | -0,105 | -0,105 |  | -0,138 | -0,138 |  | -0,124 | -0,124 |  |
| Length | -0,080 | -0,006 |  | -0,012 | 0,072 |  | 0,196 | 0,242 |  | 0,191 | 0,222 |  | 0,256 | 0,282 |  | 0,130 | 0,200 |  |
| Weight | -0,029 | -0,029 |  | 0,018 | 0,018 |  | **0,230*** | **0,230*** | **0,053*** | **0,251*** | **0,251*** | **0,063*** | **0,342*** | **0,342*** | **0,117*** | 0,191 | 0,191 |  |
| BSA | -0,022 | -0,022 |  | 0,035 | 0,035 |  | **0,245*** | **0,245*** | **0,060*** | **0,254*** | **0,254*** | **0,065*** | **0,334*** | **0,334*** | **0,111*** | 0,203 | 0,203 |  |
| Dose | 0,143 | 0,143 |  | 0,140 | 0,140 |  | **0,533**** | **0,533**** | **0,284**** | **0,522**** | **0,522**** | **0,272**** | **0,448**** | **0,448**** | **0,201**** | **0,451**** | **0,203**** | **0,204**** |
| Dose/BSA | 0,170 | 0,170 |  | 0,150 | 0,150 |  | **0,475**** | **0,475**** | **0,226**** | **0,433**** | **0,433**** | **0,188**** | 0,267 | 0,267 |  | **0,389**** | **0,451**** | **0,151**** |
| Duration of treatment | 0,023 | -0,049 |  | 0,070 | 0,114 |  | 0,177 | 0,124 |  | 0,141 | 0,068 |  | 0,026 | 0,037 |  | 0,174 | 0,086 |  |
| Route of administration | -0,142 | -0,142 |  | -0,176 | -0,176 |  | 0,075 | 0,075 |  | 0,137 | 0,137 |  | 0,192 | 0,192 |  | 0,001 | 0,001 |  |
